# Questionnaire Survey of Japanese Patients With Inflammatory Bowel Disease and Physicians on Shared Decision-Making in Advanced Therapy: A Web-Based PAIR Survey

**DOI:** 10.1093/crocol/otaf014

**Published:** 2025-04-09

**Authors:** Fumihito Hirai, Takayuki Matsumoto, Keita Imai, Yuki Goda, Yuki Fujimitsu, Toshifumi Kajioka, Masami Oiwa, Tomoki Honjo, Masaaki Higashikawa, Masato Ueno

**Affiliations:** Department of Gastroenterology, Faculty of Medicine, Fukuoka University, Fukuoka, Japan; Division of Gastroenterology, Department of Internal Medicine, School of Medicine, Iwate Medical University, Iwate, Japan; Department of IBD Strategy, EA Pharma Co., Ltd., Tokyo, Japan; Department of IBD Strategy, EA Pharma Co., Ltd., Tokyo, Japan; Department of IBD Strategy, EA Pharma Co., Ltd., Tokyo, Japan; Department of IBD Strategy, EA Pharma Co., Ltd., Tokyo, Japan; Department of IBD Strategy, EA Pharma Co., Ltd., Tokyo, Japan; Department of IBD Strategy, EA Pharma Co., Ltd., Tokyo, Japan; Department of Pharmaceutical Development, Development Promotion & Data Science Group, EA Pharma Co., Ltd., Tokyo, Japan; Department of IBD Strategy, EA Pharma Co., Ltd., Tokyo, Japan

**Keywords:** ulcerative colitis, Crohn’s disease, inflammatory bowel disease, preference, shared decision-making

## Abstract

**Background/Aims:**

With the recent increase in available treatment options for inflammatory bowel disease (IBD), shared decision-making has gained considerable importance. To address potential disparities in patient and physician priorities, we conducted a survey to clarify these perspectives.

**Methods:**

Patients with IBD and physicians treating IBD were asked to complete an online questionnaire focused on key factors influencing drug selection and preferred drug administration methods.

**Results:**

Responses were obtained from 400 patients (327 with ulcerative colitis and 73 with Crohn’s disease) and 155 physicians. Among the factors in drug selection, physicians assigned significantly higher importance scores for experience with the drug than did patients. The expected time to onset of drug effects was significantly different between patients and physicians. Regarding preferences for drug administration method, patients and physicians assigned the highest acceptability scores for once-daily oral administration. For intravenous and subcutaneous routes, patients’ scores were significantly lower than those of physicians’ scores. Notably, 86.0% of patients and 62.0% of physicians preferred oral administration as the most preferred method. However, preferences varied based on treatment experience: 34.7% of patients with prior experience with subcutaneous injection preferred this method.

**Conclusions:**

Patients and physicians generally shared similar priorities for drug selection; however, physicians emphasized their experience with the drug over patient preferences. Although the number of patients with prior treatment experience preferred intravenous or subcutaneous injections, oral formulations remained the preferred choice for both patients and physicians.

## Graphical Abstract

**Graphical Abstract:**
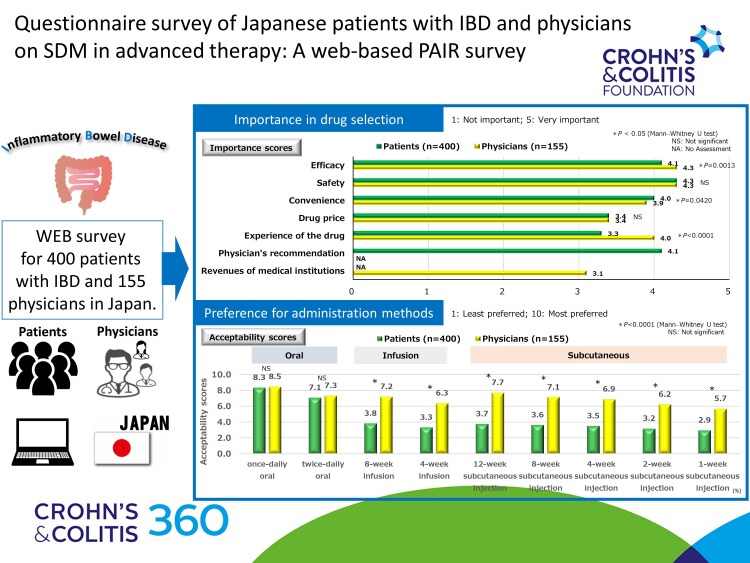

## Introduction

Inflammatory bowel disease (IBD), which includes ulcerative colitis (UC) and Crohn’s disease (CD), is a chronic condition of unknown etiology.^1^ Patients with IBD experience diarrhea, abdominal pain, fever, weight loss, and bloody stools, which considerably impaired their quality of life.^2^ Although the prevalence of IBD varies by region and country, IBD has been increasing in recent years, due in part to environmental changes, dietary changes, and genetic predisposition.^3,4^ Current treatment options for refractory IBD include antitumor necrosis factor antibody, anti-p40 antibody, anti-p19 antibody, anti-α4β7 integrin antibody, janus kinase inhibitors, and sphingosine 1-phosphate receptor modulators.^5–15^ These drugs can be administered through various routes, including oral, intravenous, or subcutaneous, in differing frequencies. Although guidelines have been provided for the treatment of IBD, there are many therapeutic agents and not all of them are clear on how to choose.^16–19^

Patients and physicians often differ in their treatment priorities, underscoring the need for shared decision-making (SDM).^20,21^ When selecting a drug, in addition to efficacy, safety, and drug information, patient acceptability should also be considered.^22,23^ However, patient and physician perceptions may not always align, which can impact treatment adherence and quality of the doctor-patient relationship. To better understand these perspectives, we conducted the PAIR survey (Patients with IBD and doctors), an internet-based survey to explore the priorities of both patients and physicians in drug selection and convenience of administration methods.

## Methods

### Survey Instruments

Surveys were conducted by the Cross Marketing Corporation (Tokyo, Japan) targeting both IBD patients and physicians treating IBD. The survey of IBD patients was conducted between August 2 and 8, 2024, inviting 492 IBD patients nationwide (403 with UC, 89 with CD) to participate in an online questionnaire survey. Among them, 400 patients (327 with UC, 73 with CD) agreed to participate in the survey and consented to the disclosure of results. The physician’s survey was conducted online from August 8 to 9, 2024, inviting all specialties 33 257 physicians nationwide, and 155 physicians who treated at least 5 patients with IBD responded.

The survey questionnaire for patients included the following items: type of disease (UC/CD), disease duration of IBD, smoking status, occupation, history of bowel resection, history of hospitalization, type of attending institution, severity of symptoms (degree of diarrhea and abdominal pain on a 4-point scale of none/ mild/ moderate/ severe), current treatment methods, experience with drug selection, importance of efficacy, safety, convenience of administration, cost of the drug, experience with the drug, physician’s recommendation in drug selection (importance scores rated on a scale of 1–5), expected time to onset of drug effects, preference of administration method (acceptability scores on a scale of 1–10), the most preferred method of administration, evaluation of the usefulness oral administration (on a scale of 1–10), most perceived benefit of oral administration. The survey items for physicians included age, sex, type of medical institution and department, number of patients treated for UC and CD, criteria for selecting advanced therapies, importance of efficacy, safety, convenience of administration, cost of the drug, experience with the drug, and revenues of medical institutions in drug selection (importance scores on a scale of 1–5), expected time to onset of drug effects, preference for administration methods (acceptability scores on a scale of 1–10), the most preferred method of administration, and evaluation of the usefulness of oral administration (on a scale of 1–10), and the advantages of oral administration. Informed consent from both patients and physicians was obtained online for conducting and publishing the results of the survey.

### Outcome

The primary endpoint was the comparison of acceptability scores from 1 (least preferred) to 10 (most preferred) for the 9 drug administration methods and the differences in these scores between patients and physicians. Secondary endpoints included the most preferred drug administration method, importance scores for various drug selection factors from 1 (not important) to 5 (very important), evaluation of the usefulness of oral administration scores from 1 (not good) to 10 (very good), most useful merit of oral administration, expected time to onset of drug effect, and the difference between patients’ and physicians’ responses.

### Statistical Analysis

All statistical analyses were performed using the SAS software (version 9.4; SAS Institute Japan Ltd.). Statistical analysis was conducted using the Pearson chi-square test for categorical data and the Mann–Whitney U test or Wilcoxon signed-rank test for continuous variables. A *P*-value < .05 was considered statistically significant.

## Results

### Demographics of the Patients and Physicians

Responses were received from 400 patients (327 with UC, 73 with CD) and 155 physicians, and all patients and physicians who responded were included in this questionnaire survey. All responses were eligible and included in the analysis. The mean age of IBD patients was 53.1 years, mean duration of disease was 16.0 years, and 66.5% of the patients were men (Table 1). Occupational status was full-time (50.8%), part-time (16.5%), student (0%), housework (8.8%), other (4.8%), and unemployed (19.3%). Diarrhea, abdominal pain, and other subjective symptoms were not present in 42.5% of the patients, mild in 40.3%, moderate in 15.5%, and severe in 1.8%. The mean age of the physicians was 49.3 years, 99.5% were men, and 61.3% were gastroenterologists (Table 2). The average number of patients treated per physician was 23.7 UC patients and 14.1 CD patients.

**Table 1.** Demographic characteristics of the patients.

Number of patients	400
UC	81.8% (327/400)
CD	18.3% (73/400)
Sex (male)	66.5% (266/400)
Age (mean ± SD)	53.1 ± 11.2 years
Disease duration (mean ± SD)	16.0 ± 10.8 years
Working status	
Full-time	50.8% (203/400)
Part-time	16.5% (66/400)
Student	0% (0/400)
Housework	8.8% (35/400)
Other	4.8% (19/400)
Unemployed	19.3% (77/400)
Smoking	
Never	49.5% (198/400)
Present	16.8% (76/400)
Past	33.8% (135/400)
History of resection	16.5% (66/400)
Number of hospitalizations (mean ± SD)	2.2 ± 5.2
Severity of current symptoms	
None	42.5% (170/400)
Mild	40.3% (161/400)
Moderate	15.5% (62/400)
Severe	1.8% (7/400)
Visiting institutions	
University hospital	21.0% (84/400)
General hospital	52.8% (211/400)
Clinic	25.8% (103/400)
No regular visits	0.5% (2/400)
Treatment history	
Oral therapy	96.3% (385/400)
Nutritional therapy	19.3% (77/400)
Infusion or injection therapy	34.0% (136/400)
Self-injection therapy	12.3% (49/400)
Topical therapy	46.8% (187/400)
Other	2.3% (9/400)
None	1.3% (5/400)

CD, Crohn’s disease; UC, ulcerative colitis.

**Table 2.** Physicians’ demographic characteristics.

Number of physicians	155
Sex (male)	95.5% (148/155)
Age (mean ± SD)	49.3 ± 10.0 years
Medical institution	
University hospital	21.3% (33/155)
General hospital	61.9% (96/155)
Clinic	16.8% (26/155)
Specialization	
Gastroenterology	61.3% (95/155)
Surgery	11.6% (18/155)
General physician	25.2% (39/155)
Other	1.9% (3/155)
Number of patients treated	
IBD	37.8 ± 57.7
UC	23.7 ± 38.5
CD	14.1 ± 21.1

CD, Crohn’s disease; IBD, inflammatory bowel disease; UC, ulcerative colitis.

### Experience and Method of Drug Selection

Among the patients, 34.5% reported that their physicians had presented them with multiple drug options, allowing them to make the final choice. Regarding the physicians’ approach to drug selection, 18.2% indicated that they presented all the drug options and let the patient make the decision. In contrast, 54.1% indicated that the physician narrowed down the available drug options and let the patient make the decision, and 27.8% indicated that the physician chose the drug (Figure S1).

### Importance in Drug Selection

**Figure 1.**
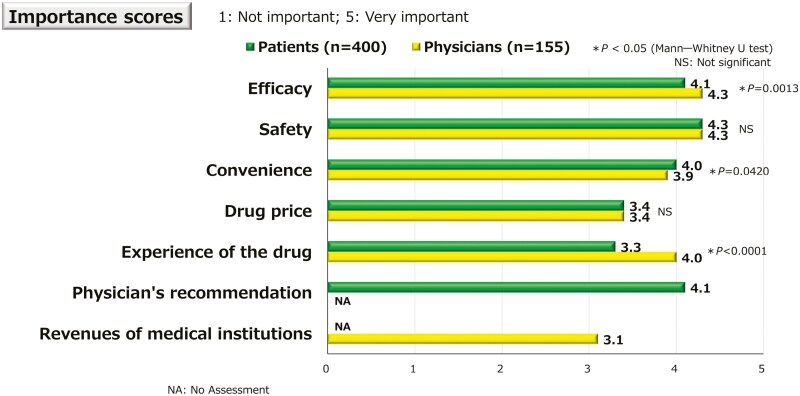
Importance in drug selection. The 5-level importance scores in drug selection between patients and physicians. Although the differences between patients’ and physicians’ scores were not large, patients assigned significantly lower importance scores for efficacy and higher importance scores for convenience than those of physicians (*P* = .0013 and *P* = .0420, respectively). Compared with patients, physicians had significantly higher importance scores for experience with the drug (*P *< .0001).

In drug selection, the importance scores for safety and drug cost were not significantly different between patients and physicians. However, patients assigned significantly lower importance scores for efficacy and higher importance scores for convenience than did physicians, although the differences were not so large (*P *= .0013 and *P *= .0420, respectively). Compared with patients, physicians assigned significantly higher importance scores for experience with the drug (*P *< .0001) (Figure 1).

### Expected Time to Onset of Drug Effect

**Figure 2.**
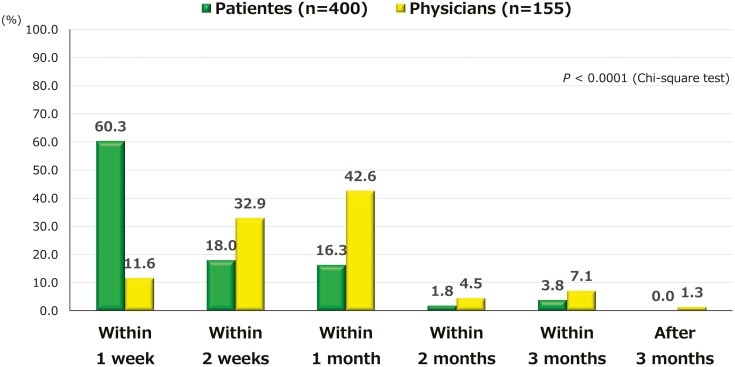
Expected time to onset of drug effect. The expected time to onset of drug effects was significantly different between patients and physicians (*P *< .0001).

Regarding expected time to onset of drug effect, a significant difference was observed between the 2 groups (*P *< .0001). Among the physicians, 42.6% expected the drug to be effective within 1 month and 11.6% within 1 week, whereas 60.3% of patients expected the drug to be effective within 1 week (Figure 2).

### Preference for Administration Methods

**Figure 3.**
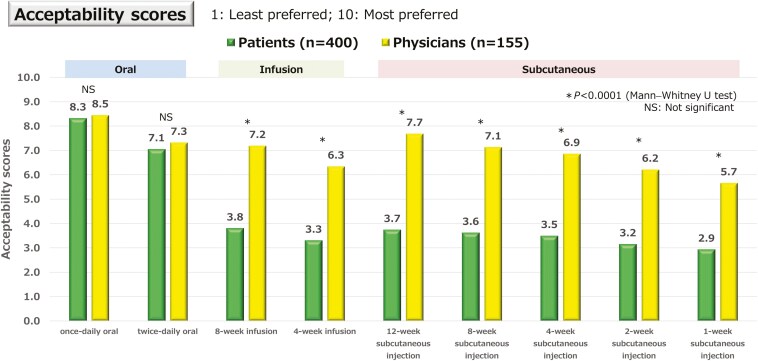
Preference for administration methods. The 10-level acceptability scores for administration methods. Acceptability scores were higher for once-daily oral administration, with significant differences between other administration methods in both patients and physicians (*P* <.0001). Patients’ scores were significantly lower than those of physicians’ scores for intravenous and subcutaneous methods of administration (*P *< .0001).

**Figure 4.**
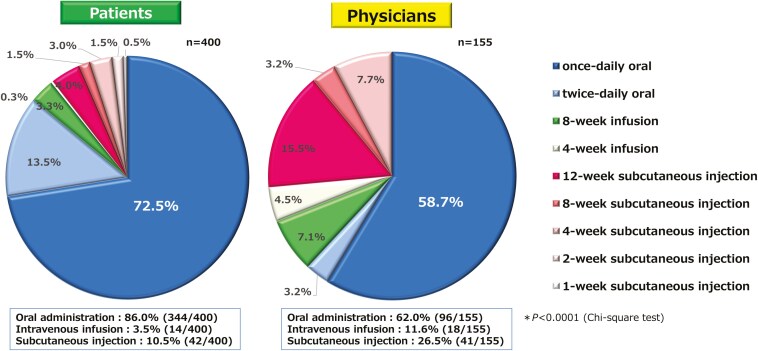
Most preferred method of administration. Compared with patients, physicians were significantly less likely to consider oral administration as the most preferred administration method (*P *< .0001).

Among patients, once-daily oral administration had the highest acceptability score, with significant differences between oral administration twice daily and intravenous infusion at each dosing interval, and subcutaneous injection at each dosing interval (*P* <.0001) (Figure 3). Similarly, physicians also assigned the highest acceptability scores for once-daily oral administration, with significant differences between oral administration twice daily, intravenous infusion at each dosing interval, and subcutaneous injection at each dosing interval (*P* <.0001). For intravenous infusions or subcutaneous injections, patients with prior experience with these methods assigned higher scores than those with no prior experience; however, the once-daily oral formulation remained the most preferred method with highest acceptability scores (Figure S2). The acceptability scores for oral treatment were similar between patients and physicians, whereas patients’ scores for intravenous and subcutaneous routes were significantly lower than those of physicians’ scores. Regarding the most preferred method of administration, 72.5% of patients preferred once-daily oral administration, and 86.0% preferred oral administration when twice daily was included. Moreover, physicians preferred once-daily oral administration, with 58.7% indicating it as their top choice, and 62.0% preferred oral administration when twice-daily dosing was included; however, this was significantly lower than those indicated by patients (*P *< .0001) (Figure 4). Results by patients’ treatment experience are shown in Figure S3.

### Usefulness of Oral Administration

**Figure 5.**
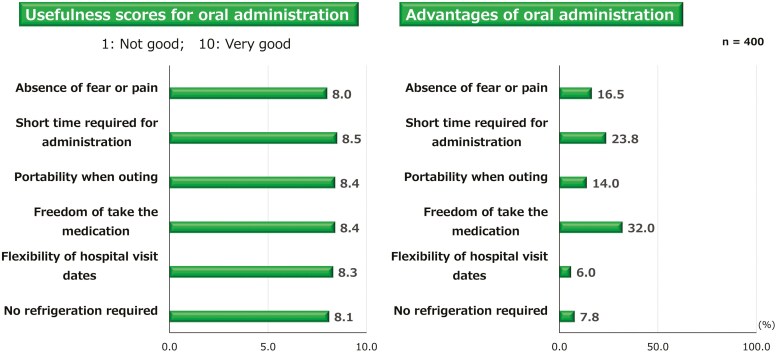
Patients’ perspective of usefulness of oral administration

**Figure 6.**
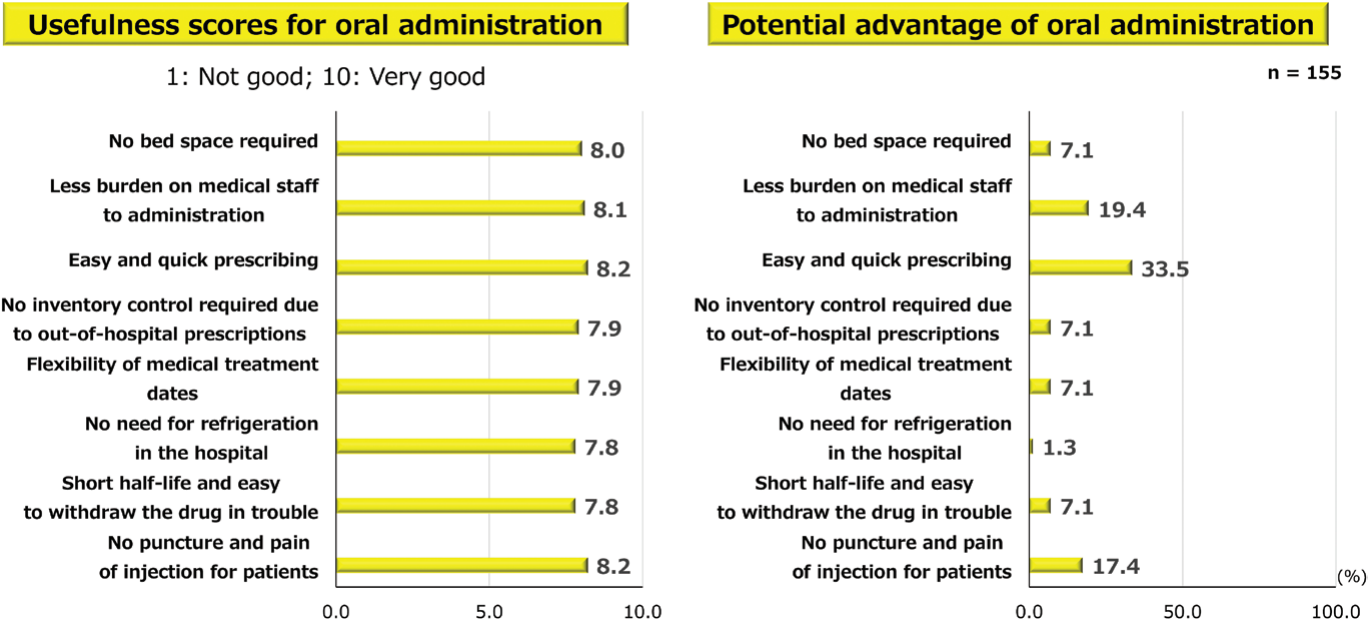
Physicians’ perspective of usefulness of oral administration

In the patients’ evaluation of the usefulness of oral administration, high scores were given for several items. The most valued advantage was the “freedom to take the medication at any time and place” (32.0%), followed by “short time required for administration” (23.8%), and the “absence of injection-related fear or pain” (16.5%) (Figure 5). Similarly, oral administration was rated high by the physicians for several reasons, including ease and quick prescription (33.5%), less burden on medical staff for administering the medication (19.4%), and the avoidance of puncture or injection-induced pain for patients (17.4%) (Figure 6).

## Discussion

Although mutual agreement between physicians and patients is crucial in treating IBD, a gap often exists between the perceptions of physicians and patients.^24–26^ With the development of new drugs that can be administered orally, intravenously, or subcutaneously at different frequencies,^5–15^ SDM has become increasingly important in the treatment of IBD.^20,21^ Effective drug selection requires consideration of efficacy, safety, and drug information, in addition to patient acceptability.^22,23^ This online-based survey aimed to investigate both patients’ and physicians’ priorities when selecting advanced therapies for IBD and their preferences for administration methods. The low physician response rate is due to the fact that the survey panel was registered from all specialties, and only physicians with experience with IBD responded to the survey.

Given the challenges in predicting the efficacy and adverse effects of IBD treatments, SDM is a beneficial approach for selecting a drug from among various advanced therapies. However, with the increasing number of available advanced therapies, time constraints make it difficult to present all options. Notably, our survey results indicated that only 18.2% of physicians indicated that they presented all available drug options, whereas 54.1% of physicians narrowed down the list. While the traditional paternalistic approach of the doctor-patient relationship is decreasing, SDM is being implemented in a more practical and time-efficient manner in real clinical practice. This study highlights that SDM, which is applied as an approach known as “libertarian paternalism,” is being used in clinical practice. In the past, the choice of treatment was generally based on the so-called paternalism, in which the patient accepts the treatment proposed by the doctor. However, SDM is now recommended, and doctors are expected to support patients’ decision-making as the expert while respecting their preferences and free will.^27^ On the other hand, in cases where there are many treatment options, such as in the field of IBD, simply presenting a lot of options can often confuse patients and make it more difficult for them to choose.^28^ To solve this problem of difficulty in choosing a treatment, the method of “libertarian paternalism” is thought to be effective. Libertarian paternalism is a concept that is a combination of paternalism and libertarianism.^29^ For example, narrowing down the options and recommending them from many treatment options is thought to be a concrete example of libertarian paternalism. In that case, it is important to consider the patient’s lifestyle and preferences, rather than just the stereotypical patient’s medical condition.

Regarding the importance of each item in patient and physician drug selection, patients and physicians had assigned similar importance scores for efficacy, safety, convenience, and drug cost; however, physicians assigned significantly higher importance scores for experience with the drug than did patients. This suggests that physicians may emphasize their experience with certain drugs. Regarding the expected time to onset of the drug effect, 60% of patients expected the drug to be effective within 1 week, whereas only 12% of physicians anticipated this outcome. The percentage of patients expected the drug to be effective within 1 week, which aligns with the results of a survey on patients with rheumatoid arthritis.^30,31^ Recently, there have been an increasing number of study reports about early efficacy, but realistically, efficacy rates in the 1 week or 2 weeks after administration are never as high as patients would like.^32–36^ The lower percentage of physicians expecting the drug to be effective within 1 week may reflect a perceived practical difficulty. Nonetheless, this represents a gap in expectations, as most patients expect quick relief. To better align with IBD patients’ needs, physicians should consider earlier symptom improvement wherever feasible. The expected time to efficacy may differ depending on the severity of symptoms, but no notable differences were observed, due in part to the small number of patients with moderate or severe symptoms in this study.

Among the 9 administration methods evaluated, patients assigned the highest acceptability scores for once-daily oral administration, with 86.0% of patients preferring oral administration, which aligns with that of previous studies.^37–40^ Patients with prior experience with subcutaneous and intravenous formulations showed improved acceptance to these methods,^38,41^ and patients with prior treatment also assigned higher scores for these formulations; however, the oral formulation remained the most preferred choice with the highest score, which is consistent with the results of a previous survey conducted in Japan.^40^ We also evaluated by severity of symptoms, but no notable differences were observed. Additionally, this survey evaluated physicians’ preferences for drug administration methods. We believe that this is the first study to explore physicians’ preferences of drug administration for advanced therapies in IBD. The acceptability scores for oral administration were comparable between the 2 groups; however, patients’ assigned significantly lower scores for intravenous and subcutaneous routes than did physicians. Furthermore, compared with patients, physicians about the most preferred method of administration were significantly less likely to prefer oral administration, suggesting that physicians may overestimate the convenience of intravenous and subcutaneous formulations. Subcutaneous injections and infusions are also accepted by patients in daily practice, and we assume that physicians consider subcutaneous injections and infusions to be more convenient because of the longer dosing intervals than oral agents. Though the drug selection is based not only on the method of administration, but it is important to carefully consider patients’ opinions regarding the acceptability of the administration method when implementing SDM.

This study had several limitations. First, online-based survey may have introduced the possibility of a biased population. Specifically, there is concern that the elderly and other patient groups that are not comfortable with digital devices may be excluded. However, the average age of the patients in this study was 53.1 years, and there were no trends that elderly patients were specifically excluded from the study compared to the general population. Although the proportion of male patients was relatively high, this aligns closely with the general sex ratio and age distribution of patients with IBD in Japan and Asian countries.^42–45^ In addition, the regions of residence of the respondents were less biased, suggesting that geographic bias was relatively small. Second, because this was an Internet-based survey, there may be limitations in response accuracy. However, Kelstrup et al. after matching 197 patients’ Internet survey responses with their actual medical records, they report that Internet surveys can be conducted with a high degree of reliability.^46^ Furthermore, similar methodologies have been employed successfully in several other studies.^2,39,40,47^ Third, this survey was conducted only in Japan, and the results may be different in other countries. Although this is a valid concern, many sub-analyses of international studies to date have not shown that findings in Japan are significantly different from those in other countries.^24,26,30,31^ Although these ethnic variations may impact future surveys, we believe that the results of the present study are informative.

In conclusion, our online survey results highlighted both similarities and differences in the perspectives of patients with IBD and physicians treating IBD in strategizing treatment decisions and their preferences for administration methods. Overall, patients and physicians aligned on several aspects of drug selection, although physicians emphasized their previous experience with the drugs. Both patients and physicians found oral formulations to be a convenient treatment option; however, physicians may overestimate the convenience of intravenous and subcutaneous formulations over patients. These findings suggest that implementing SDM that incorporates the needs and preferences of IBD patients for selecting advanced therapies would improve patient satisfaction and adherence.

## Supplementary Material

otaf014_suppl_Supplementary_Figures_S1-S3

## Data Availability

Data not publicly available. The data used and analyzed in this study are available from the corresponding author upon reasonable request.

## References

[1] Cosnes J , Gower-Rousseau C, Seksik P, Cortot A. Epidemiology and natural history of inflammatory bowel diseases. Gastroenterology. 2011;140(6):1785-1794. doi: https://doi.org/10.1053/j.gastro.2011.01.055 21530745

[2] Matsumoto T , Yanai S, Toya Y, Ueno M, Nakamura S. Internet-orientated assessment of QOL and actual treatment status in Japanese patients with inflammatory bowel disease: the 3I survey. J Crohns Colitis. 2015;9(6):477-482. doi: https://doi.org/10.1093/ecco-jcc/jjv052 25814388

[3] Ng SC , HY S 2, Hamidi N, et al Worldwide incidence and prevalence of inflammatory bowel disease in the 21st century: a systematic review of population-based studies. Lancet. 2017;23(10114):2769-2778. doi: https://doi.org/10.1016/S0140-6736(17)32448-0. 29050646

[4] Murakami Y , Nishiwaki Y, Oba MS, et al Estimated prevalence of ulcerative colitis and Crohn’s disease in Japan in 2014: an analysis of a nationwide survey. J Gastroenterol. 2019;54(12):1070-1077. doi: https://doi.org/10.1007/s00535-019-01603-8 31309327

[5] Rutgeerts P , Sandborn WJ, Feagan BG, et al Infliximab for induction and maintenance therapy for ulcerative colitis. N Engl J Med. 2005;353(23):2462-2476. doi: https://doi.org/10.1056/NEJMoa050516 16339095

[6] Sandborn WJ , van Assche G, Reinisch W, et al Adalimumab induces and maintains clinical remission in patients with moderate-to-severe ulcerative colitis. Gastroenterology. 2012;142(2):257-265. doi: https://doi.org/10.1053/j.gastro.2011.10.032 22062358

[7] Sandborn WJ , Feagan BG, Marano C, et al; PURSUIT-SC Study Group. Subcutaneous golimumab induces clinical response and remission in patients with moderate-to-severe ulcerative colitis. Gastroenterology. 2014;146(1):85-95; quiz e14. doi: https://doi.org/10.1053/j.gastro.2013.05.048 23735746

[8] Sands BE , Sandborn WJ, Panaccione R, et al; UNIFI Study Group. Ustekinumab as induction and maintenance therapy for ulcerative colitis. N Engl J Med. 2019;381(13):1201-1214. doi: https://doi.org/10.1056/NEJMoa1900750 31553833

[9] Feagan BG , Rutgeerts P, Sands BE, et al; GEMINI 1 Study Group. Vedolizumab as induction and maintenance therapy for ulcerative colitis. N Engl J Med. 2013;369(8):699-710. doi: https://doi.org/10.1056/NEJMoa1215734 23964932

[10] Sandborn WJ , Su C, Sands BE, et al; OCTAVE Induction 1, OCTAVE Induction 2, and OCTAVE Sustain Investigators. Tofacitinib as induction and maintenance therapy for ulcerative colitis. N Engl J Med. 2017;376(18):1723-1736. doi: https://doi.org/10.1056/NEJMoa1606910 28467869

[11] Feagan BG , Danese S, Loftus EV, Jr, et al Filgotinib as induction and maintenance therapy for ulcerative colitis (SELECTION): a phase 2b/3 double-blind, randomised, placebo-controlled trial. Lancet. 2021;397(10292):2372-2384. doi: https://doi.org/10.1016/S0140-6736(21)00666-8 34090625

[12] Danese S , Vermeire S, Zhou W, et al Upadacitinib as induction and maintenance therapy for moderately to severely active ulcerative colitis: results from three phase 3, multicentre, double-blind, randomised trials. Lancet. 2022;399(10341):2113-2128. doi: https://doi.org/10.1016/S0140-6736(22)00581-5 35644166

[13] Sandborn WJ , Feagan BG, D’Haens G, et al; True North Study Group. Ozanimod as induction and maintenance therapy for ulcerative colitis. N Engl J Med. 2021;385(14):1280-1291. doi: https://doi.org/10.1056/NEJMoa2033617 34587385

[14] Sandborn WJ , Vermeire S, Peyrin-Biroulet L, et al Etrasimod as induction and maintenance therapy for ulcerative colitis (ELEVATE): two randomised, double-blind, placebo-controlled, phase 3 studies. Lancet. 2023;401(10383):1159-1171. doi: https://doi.org/10.1016/S0140-6736(23)00061-2 36871574

[15] Louis E , Schreiber S, Panaccione R, et al; INSPIRE and COMMAND Study Group. Risankizumab for ulcerative colitis: two randomized clinical trials. JAMA. 2024;332(11):881-897. doi: https://doi.org/10.1001/jama.2024.12414 39037800 PMC11264075

[16] Singh S , Loftus EV, Jr, Limketkai BN, et al; AGA Clinical Guidelines Committee. Electronic address: clinicalpractice@gastro.org. AGA living clinical practice guideline on pharmacological management of moderate-to-severe ulcerative colitis. Gastroenterology. 2024;167(7):1307-1343. doi: https://doi.org/10.1053/j.gastro.2024.10.001 39572132 PMC12162142

[17] Feuerstein JD , Ho EY, Shmidt E, et al; American Gastroenterological Association Institute Clinical Guidelines Committee. AGA clinical practice guidelines on the medical management of moderate to severe luminal and perianal fistulizing Crohn’s disease. Gastroenterology. 2021;160(7):2496-2508. doi: https://doi.org/10.1053/j.gastro.2021.04.022 34051983 PMC8988893

[18] Raine T , Bonovas S, Burisch J, et al ECCO guidelines on therapeutics in ulcerative colitis: medical treatment. J Crohns Colitis. 2022;16(1):2-17. doi: https://doi.org/10.1093/ecco-jcc/jjab178 34635919

[19] Gordon H , Minozzi S, Kopylov U, et al ECCO guidelines on therapeutics in Crohn’s disease: medical treatment. J Crohns Colitis. 2024;18(10):1531-1555. doi: https://doi.org/10.1093/ecco-jcc/jjae091 38877997

[20] Siegel CA. Shared decision making in inflammatory bowel disease: helping patients understand the tradeoffs between treatment options. Gut. 2012;61(3):459-465. doi: https://doi.org/10.1136/gutjnl-2011-300988 22187072

[21] Mahlich J , Matsuoka K, Sruamsiri R. Shared decision making and treatment satisfaction in Japanese patients with inflammatory bowel disease. Dig Dis. 2017;35(5):454-462. doi: https://doi.org/10.1159/000471795 28380481

[22] Patel DB , van Deen WK, Almario CV, et al Assessing patient decision-making on biologic and small-molecule therapies in inflammatory bowel diseases: insights from a conjoint analysis in the United States, Canada, and the United Kingdom. Inflamm Bowel Dis. 2021;27(10):1593-1601. doi: https://doi.org/10.1093/ibd/izaa311 33300555

[23] Gisbert JP , Schreiber S, Siegel CA, et al Benefit-risk trade-offs and patient preferences for therapy selection in ulcerative colitis: a multicountry preference study. Inflamm Bowel Dis. 2024. published online Aug 10. doi: https://doi.org/10.1093/ibd/izae162 PMC12069987 39126434

[24] Rubin DT , Hart A, Panaccione R, et al Ulcerative Colitis Narrative global survey findings: communication gaps and agreements between patients and physicians. Inflamm Bowel Dis. 2021;27(7):1096-1106. doi: https://doi.org/10.1093/ibd/izaa257 33057598 PMC8214018

[25] Dubinsky MC , Watanabe K, Molander P, et al Ulcerative Colitis Narrative Global survey findings: the impact of living with ulcerative colitis - a patients’ and physicians’ view. Inflamm Bowel Dis. 2021;27(11):1747-1755. doi: https://doi.org/10.1093/ibd/izab016 33529314 PMC8528151

[26] Watanabe K , Gardiner S, Arai S. Notable gaps between patients’ and physicians’ perspectives on communication and disease management in Japan: multifaceted ad hoc analyses of the global Ulcerative Colitis Narrative Survey for further optimal care. Therap Adv Gastroenterol 2022;15. published online Jun 14. doi: https://doi.org/10.1177/17562848221095372 PMC9201355 35721839

[27] Barry MJ , Edgman-Levitan S. Shared decision making--pinnacle of patient-centered care. N Engl J Med. 2012;366(9):780-781. doi: https://doi.org/10.1056/NEJMp1109283 22375967

[28] Sunstein CR. Choosing not to choose. Duke Law J. 2014;64:1-52. 25330554

[29] Sunstein CR , Thaler R. Libertarian paternalism is not an oxymoron. Univ Chicago Law Rev 2003;70(4):1159-1202.

[30] Taylor PC , Ancuta C, Nagy O, et al Treatment satisfaction, patient preferences, and the impact of suboptimal disease control in a large international rheumatoid arthritis cohort: SENSE Study. Patient Prefer Adherence 2021;15:359-373. doi: https://doi.org/10.2147/PPA.S289692 33633444 PMC7900444

[31] Kawahito Y , Takakubo Y, Morinobu A, Matsubara N, Nagy O, Sugiyama E. Patient satisfaction, preferences, expectations, characteristics, and impact of suboptimal control of rheumatoid arthritis: a subgroup analysis of Japanese patients from a large international cohort study (SENSE). PLoS One. 2021;16(11):e0259389. published online Nov 15. doi: https://doi.org/10.1371/journal.pone.0259389 34780502 PMC8592402

[32] Ahuja D , Murad MH, Ma C, Jairath V, Singh S. Comparative speed of early symptomatic remission with advanced therapies for moderate-to-severe ulcerative colitis: a systematic review and network meta-analysis. Am J Gastroenterol. 2023;118(1):1618-1625. doi: https://doi.org/10.14309/ajg.0000000000002263 36976548

[33] Hanauer S , Panaccione R, Danese S, et al Tofacitinib induction therapy reduces symptoms within 3 days for patients with ulcerative colitis. Clin Gastroenterol Hepatol. 2019;17(1):139-147. doi: https://doi.org/10.1016/j.cgh.2018.07.009 30012431

[34] Danese S , Ferrante M, Feagan BG, et al Rapid and sustained symptom relief in patients with ulcerative colitis treated with filgotinib: data from the Phase 2b/3 SELECTION Trial. Am J Gastroenterol. 2023;118(1):138-147. doi: https://doi.org/10.14309/ajg.0000000000001979 36113491 PMC9810009

[35] Loftus EV, Jr, Colombel JF, Takeuchi K, et al Upadacitinib therapy reduces ulcerative colitis symptoms as early as day 1 of induction treatment. Clin Gastroenterol Hepatol. 2023;21(9):2347-2358.e6. doi: https://doi.org/10.1016/j.cgh.2022.11.029 36464141

[36] Danese S , Dignass A, Matsuoka K, et al Early and sustained symptom control with Mirikizumab in patients with ulcerative colitis in the Phase 3 LUCENT Program. J Crohns Colitis. 2024;18(11):1845-1856. doi: https://doi.org/10.1093/ecco-jcc/jjae088 38869019 PMC11532612

[37] Denesh D , Carbonell J, Kane JS, Gracie D, Selinger CP. Patients with inflammatory bowel disease (IBD) prefer oral tablets over other modes of medicine administration. Expert Rev Gastroenterol Hepatol. 2021;15(9):1091-1096. doi: https://doi.org/10.1080/17474124.2021.1898944 33653185

[38] Buisson A , Serrero M, Orsat L, et al Comparative acceptability of therapeutic maintenance regimens in patients with inflammatory bowel disease: results from the Nationwide ACCEPT2 Study. Inflamm Bowel Dis. 2023;29(4):579-588. doi: https://doi.org/10.1093/ibd/izac119 35815744

[39] Matsumoto T , Imai K, Goda Y, et al Questionnaire survey for inflammatory bowel disease patients in Japan; a web-based Japan, Crohn’s disease, ulcerative colitis, patients survey. Crohns Colitis. 2023;5(4):ota9069. published online Nov 17. doi: https://doi.org/10.1093/crocol/otad069 PMC10676197 38028953

[40] Morishita T , Yanai S, Toya Y, Matsumoto T. Patients’ preference on advanced therapy and follow-up procedure for inflammatory bowel disease in Japan: a web-based 3A survey. Inflamm Intest Dis. 2024;9(1):174-183. doi: https://doi.org/10.1159/000539738 39144836 PMC11324213

[41] van Deen WK , Khalil C, Bonthala NN, et al Inflammatory bowel disease patients’ preferences for subcutaneous versus intravenous therapies: a mixed-methods study. Dig Dis. 2023;41(3):412-421. doi: https://doi.org/10.1159/000528586 36476714

[42] Asakura K , Nishiwaki Y, Inoue N, Hibi T, Watanabe M, Takebayashi T. Prevalence of ulcerative colitis and Crohn’s disease in Japan. J Gastroenterol. 2009;44(7):659-665. doi: https://doi.org/10.1007/s00535-009-0057-3 19424654

[43] Ng SC , Tang W, Ching JY, et al; Asia–Pacific Crohn's and Colitis Epidemiologic Study (ACCESS) Study Group. Incidence and phenotype of inflammatory bowel disease based on results from the Asia-pacific Crohn’s and colitis epidemiology study. Gastroenterology. 2013;145(1):158-165.e2. doi: https://doi.org/10.1053/j.gastro.2013.04.007 23583432

[44] Ng SC , Kaplan GG, Tang W, et al Population density and risk of inflammatory bowel disease: a prospective population-based study in 13 countries or regions in Asia-Pacific. Am J Gastroenterol. 2019;114(1):107-115. doi: https://doi.org/10.1038/s41395-018-0233-2 30177785

[45] Kim HJ , Hann HJ, Hong SN, et al Incidence and natural course of inflammatory bowel disease in Korea, 2006–2012: a nationwide population based study. Inflamm Bowel Dis. 2015;21(3):623-630. doi: https://doi.org/10.1097/MIB.0000000000000313 25647154

[46] Kelstrup AM , Juillerat P, Korzenik J. The accuracy of self-reported medical history: a preliminary analysis of the promise of internet-based research in inflammatory bowel diseases. J Crohns Colitis. 2014;8(5):349-356. doi: https://doi.org/10.1016/j.crohns.2013.09.012 24183653

[47] Yanai S , Toya Y, Nakamura S, Matsumoto T. Patients’ preference of topical therapy for ulcerative colitis in Japan: a web-based 3T survey. Crohn’s Colitis 2020;2(2):otaa030. doi: https://doi.org/10.1093/crocol/otaa030 PMC9927820 36798649

