# Supplementary material for: Questionnaire Survey of Japanese Patients With Inflammatory Bowel Disease and Physicians on Shared Decision-Making in Advanced Therapy: A Web-Based PAIR Survey
Source: Crohns Colitis 360. 2025 Apr 9;7(2):otaf014. doi: 10.1093/crocol/otaf014 (PMC12059213; doi:10.1093/crocol/otaf014)
Supplement: otaf014_suppl_Supplementary_Figures_S1-S3 [file otaf014_suppl_supplementary_figures_s1-s3.pptx]

## Slide 1
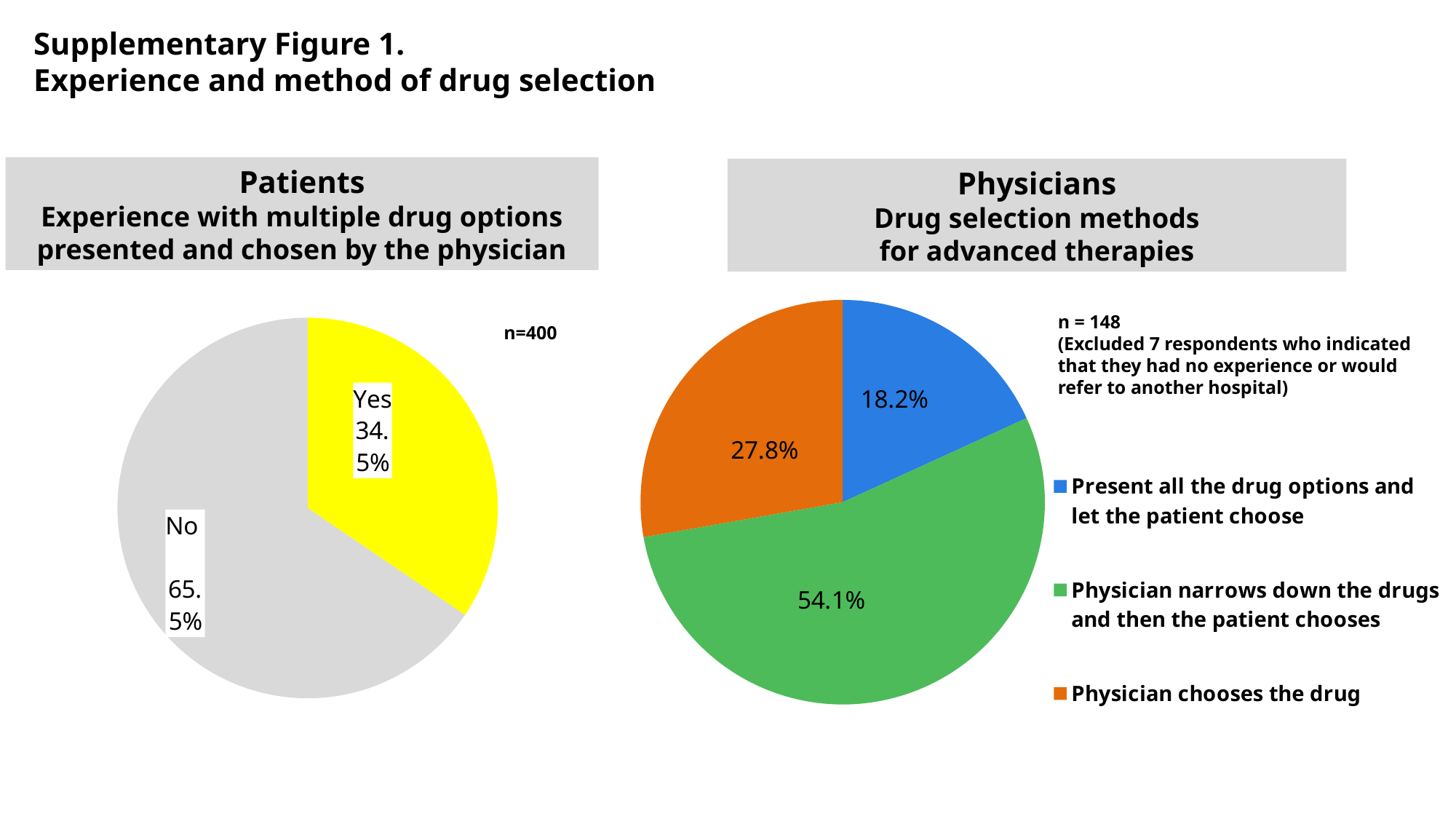

# Supplementary Figure 1. Experience and method of drug selection
Patients
Experience with multiple drug options presented and chosen by the physician
Physicians
Drug selection methods
for advanced therapies
### Chart
| Category | 系列 1 |
|---|---|
| ある | 34.5 |
| ない | 65.5 |
### Chart
| Category | 系列 1 |
|---|---|
| Present all the drug options and let the patient choose | 18.2 |
| Physician narrows down the drugs and then the patient chooses | 54.1 |
| Physician chooses the drug | 27.8 |n = 148
(Excluded 7 respondents who indicated that they had no experience or would refer to another hospital)
n=400

## Slide 2
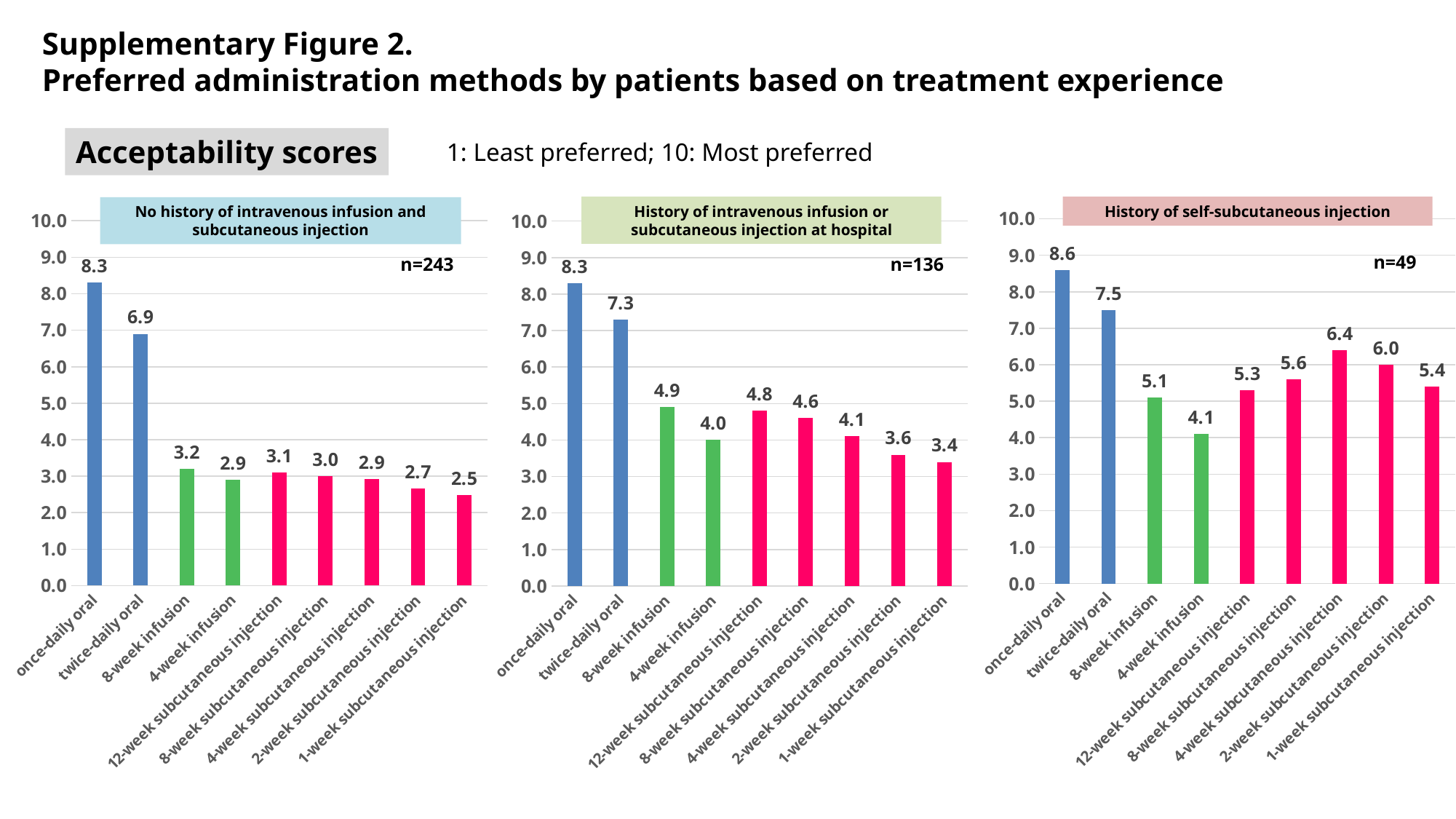

# Supplementary Figure 2. Preferred administration methods by patients based on treatment experience
Acceptability scores
1: Least preferred; 10: Most preferred
### Chart
| Category | 系列 1 |
|---|---|
| once-daily oral | 8.6 |
| twice-daily oral | 7.5 |
| 8-week infusion | 5.1 |
| 4-week infusion | 4.1 |
| 12-week subcutaneous injection | 5.3 |
| 8-week subcutaneous injection | 5.6 |
| 4-week subcutaneous injection | 6.4 |
| 2-week subcutaneous injection | 6.0 |
| 1-week subcutaneous injection | 5.4 |
### Chart
| Category | 系列 1 |
|---|---|
| once-daily oral | 8.3 |
| twice-daily oral | 6.9 |
| 8-week infusion | 3.2 |
| 4-week infusion | 2.9 |
| 12-week subcutaneous injection | 3.1 |
| 8-week subcutaneous injection | 3.0 |
| 4-week subcutaneous injection | 2.925333333333333 |
| 2-week subcutaneous injection | 2.6586666666666665 |
| 1-week subcutaneous injection | 2.482666666666667 |History of intravenous infusion or subcutaneous injection at hospital
History of self-subcutaneous injection
### Chart
| Category | 系列 1 |
|---|---|
| once-daily oral | 8.3 |
| twice-daily oral | 7.3 |
| 8-week infusion | 4.9 |
| 4-week infusion | 4.0 |
| 12-week subcutaneous injection | 4.8 |
| 8-week subcutaneous injection | 4.6 |
| 4-week subcutaneous injection | 4.1 |
| 2-week subcutaneous injection | 3.6 |
| 1-week subcutaneous injection | 3.4 |No history of intravenous infusion and subcutaneous injection
n=49
n=243
n=136

## Slide 3
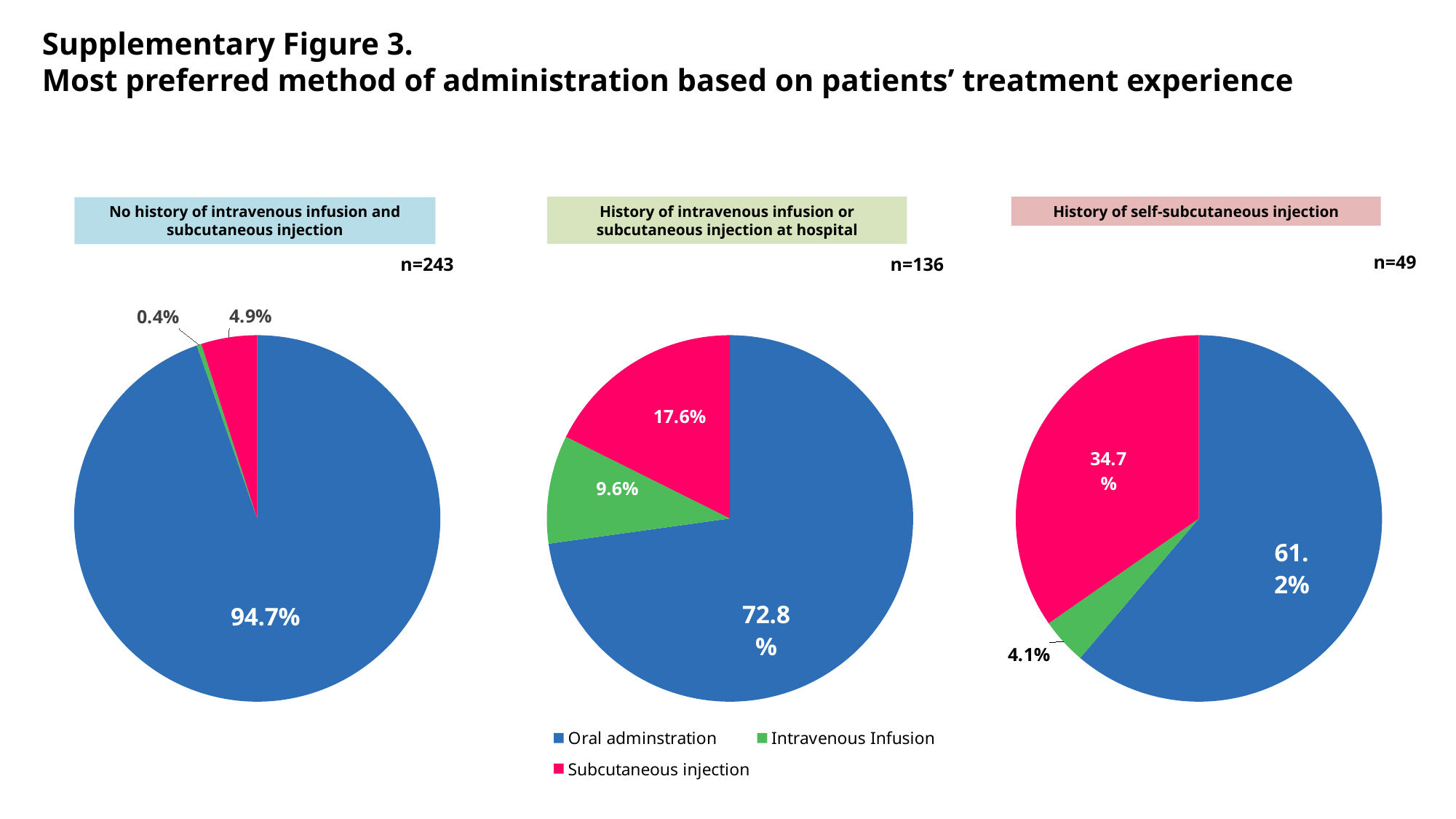

# Supplementary Figure 3. Most preferred method of administration based on patients’ treatment experience
History of intravenous infusion or subcutaneous injection at hospital
History of self-subcutaneous injection
No history of intravenous infusion and subcutaneous injection
n=49
### Chart
| Category | 系列 1 |
|---|---|
| 経口 | 230.0 |
| 点滴 | 1.0 |
| 皮下注射 | 12.0 |
### Chart
| Category | 列3 |
|---|---|
| Oral adminstration | 99.0 |
| Intravenous Infusion | 13.0 |
| Subcutaneous injection | 24.0 |
### Chart
| Category | 系列 1 |
|---|---|
| 経口 | 30.0 |
| 点滴 | 2.0 |
| 皮下注射 | 17.0 |n=243
n=136
